# Millimeter-Wave Substrate Integrated Waveguide Using Micromachined Tungsten-Coated Through Glass Silicon Via Structures

**DOI:** 10.3390/mi9040172

**Published:** 2018-04-09

**Authors:** Ik-Jae Hyeon, Chang-Wook Baek

**Affiliations:** School of Electrical and Electronics Engineering, Chung-Ang University, 84 Heukseok-ro, Dongjak-gu, Seoul 06974, Korea; everinu@hotmail.com

**Keywords:** substrate integrated waveguide (SIW), tungsten-coated through glass silicon via (TGVS), through glass via (TGV), glass reflow

## Abstract

A millimeter-wave substrate integrated waveguide (SIW) has been demonstrated using micromachined tungsten-coated through glass silicon via (TGSV) structures. Two-step deep reactive ion etching (DRIE) of silicon vias and selective tungsten coating onto them using a shadow mask are combined with glass reflow techniques to realize a glass substrate with metal-coated TGSVs for millimeter-wave applications. The proposed metal-coated TGSV structures effectively replace the metallic vias in conventional through glass via (TGV) substrates, in which an additional individual glass machining process to form micro holes in the glass substrate as well as a time-consuming metal-filling process are required. This metal-coated TGSV substrate is applied to fabricate a SIW operating at Ka-band as a test vehicle. The fabricated SIW shows an average insertion loss of 0.69 ± 0.18 dB and a return loss better than 10 dB in a frequency range from 20 GHz to 45 GHz.

## 1. Introduction

With the increased input/output (I/O) numbers of the integrated circuits (ICs), interposers with through substrate vias are essential for the small footprint, high density and low power 3-D stacking integration in advanced electronic systems. Silicon interposers using through silicon vias (TSV) have been reported numerously for the last couple of years as an alternative for conventional organic substrates because of their advantages such as ultrahigh wiring capacity, shorter signal paths with smaller parasitic effects, ease of wafer processing and die matched coefficient of thermal expansion (CTE) to the ICs [1,2]. The TSV technologies, however, have their own challenges including high fabrication cost, process complexity and large electrical substrate losses.

Recently, glass interposers based on the through glass via (TGV) technology have been extensively studied as an alternative for the silicon interposers [3,4,5,6,7,8,9,10,11,12,13,14]. Glass, as a substrate material, has several merits; closely matched CTE to silicon dies, high dimensional stability and availability in thin and large panels. Especially, the high signal isolation, low substrate loss and low material and manufacturing cost of the glass compared to conventional silicon wafers make the glass interposers attractive platforms for high frequency radio frequency (RF)/microwave passive components and packaging. Different types of RF components based on the glass interposers with TGVs such as filters [4], 3D inductors [8,9] and antennas [13,14] have been reported.

In the development of TGVs of the glass interposers, micro drilling of the glass substrate and metallization of the micro holes in the glass with conductive materials are important factors. Conventional glass drilling processes such as mechanical drilling, wet/dry etching, laser machining, sandblasting and electro discharging techniques that are currently being used for TGVs, however, are not compatible with traditional semiconductor technologies and have their own limitations in the formation of very small, fine-pitched empty via holes in the glass substrate. Complete void-free, stable via filling metallization using an electroplating process and/or sidewall metallization for the small empty via holes in the thick glass substrate are also challenging issues.

In this paper, fully micromachined tungsten-coated through glass silicon via (TGSV) structures based on the glass reflow technique have been demonstrated to realize TGVs of a glass interposer platform for RF/microwave applications. The glass substrates with silicon vias using a reflow process have already been reported in microelectromechanical systems (MEMS) community for wafer-level 3D interconnects or packaging of the microsystems [15,16,17], but its application to RF/microwave devices is limited due to the relatively low electrical conductivity of pure silicon vias compared to the metallic vias. In this work, two-step deep reactive ion etching (DRIE) of silicon vias and selective tungsten coating onto them using a shadow mask are combined with glass reflow technique to realize metal-coated TGSVs that can effectively replace the conventional metallic TGVs without any degradation of RF performances of the device. The developed tungsten-coated TGSV substrate is applied to fabricate a millimeter-wave substrate integrated waveguide (SIW) operating at Ka-band as a test vehicle.

## 2. Design and Simulation of SIW with Tungsten-Coated TGSVs

### 2.1. Structure of the SIW

SIWs have been extensively studied to demonstrate low cost, high-*Q* millimeter-wave components since they can be realized in a planar platform while keeping low loss, excellent power handling capabilities and immunity from radiation loss that classical non-planar 3D waveguides have [18,19]. These SIWs, however, are usually fabricated by using traditional microwave substrates such as printed circuit boards (PCBs) or low/high temperature cofired ceramics (LTCC/HTCCs) which are not compatible with semiconductor-based processes and hard to be integrated with silicon-based circuits or elements. For these reasons, we previously demonstrated micromachined versions of SIWs to improve integration capability of the SIW with semiconductor or MEMS devices for tunable SIW-based circuits at millimeter-wave frequencies. An SIW with gold-coated silicon vias in benzocyclobutene (BCB) polymer dielectrics was firstly reported in [20], but it suffers from the mechanical failure of the soft BCB polymer substrate material during the further high-temperature integration processes. Another SIW with electroplated copper vias in the reflowed glass dielectrics was demonstrated to increase the insensitivity of the substrate to the process temperature [21]. This work, however, needs additional removal of silicon via structures in the reflowed glass material and time-consuming seedless electroplating process which is hard to obtain completely-filled, void-free metallic copper vias with large heights.

**Figure 1:**
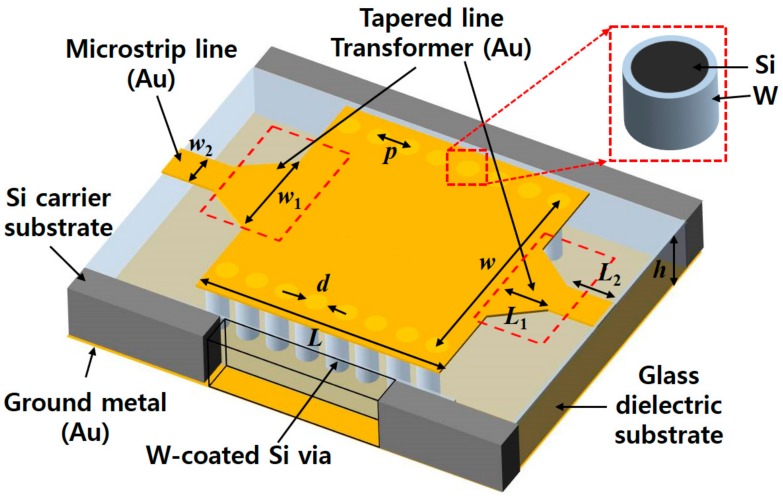
Schematic view of the proposed substrate integrated waveguide (SIW) with tungsten-coated through glass silicon via (TGSV).

In this work, SIW is demonstrated by combining the metal-coated silicon via concept and thermally reflowed glass substrate. Schematic view of the proposed SIW is illustrated in Figure 1. The SIW is composed of a glass dielectric substrate, metal-coated TGSVs and top/bottom metal layers. The dielectric substrate of the SIW is a borosilicate glass material thermally reflowed and filled into the etched trench of a low-resistive silicon wafer. The silicon wafer plays a role of a carrier substrate accepting the reflowed glass as well as a via core material. Via arrays of the SIW substituting for the sidewall of the classical rectangular waveguide are realized by the TGSV structures embedded in the glass substrate. These TGSVs are simultaneously fabricated with the silicon trench during the single-step trench forming DRIE process. Although silicon wafer with low resistivity is used, pure silicon has a conductivity four orders of magnitude smaller than conventional via metals (e.g., copper), therefore is not adequate for a via material of the SIW. For this reason, TGSVs are selectively coated with a thin metal layer using a shadow mask. Tungsten is selected as a via-metallization material because of its high melting point (3422 °C) which can help to sustain the following high-temperature glass reflow process. Top metal part of the SIW, feeding lines and transitions are formed on the top side of the substrate. Backside of the substrate is completely metallized to form a ground plane of the SIW.

### 2.2. Design and Simulation of the SIW

When the width of the waveguide is larger than the thickness of it, cut-off frequency of the dominant TE_10_ mode of the SIW is given by [22]:

(1) $$f_{c10}=\frac{c}{2w_{eff}\sqrt{\varepsilon_{r}}}$$

where $f_{c10}$ is the cut-off frequency of TE_10_ mode, *ε_r_* is the relative permittivity of the dielectric material in the waveguide, and *c* is the speed of light in vacuum. Since the continuous sidewall of the rectangular waveguide is replaced by the via arrays in the SIW, the effective width *w_eff_* is used for the SIW in Equation (1). The relationship between the effective width *w_eff_* and the center-to-center width between two rows of the vias *w* is given by [23]:

(2) $$w_{eff}=w-1.08\frac{d^{2}}{p}+0.1\frac{d^{2}}{w}$$

where *d* and *p* are the via diameter and the pitch between the vias, respectively. It is known that this modified empirical equation is accurate when *p*/*d* is smaller than 3 and *d*/*w* is smaller than 1/5 [23]. There may be radiation losses due to the energy leakage through the gaps between the vias. These radiation losses of the SIW can be maintained reasonably small if *p*/*d* < 2.5 [19].

Based on the given design criteria, the SIW with a dominant TE_10_-mode cut-off frequency of 18.6 GHz is designed and optimized using a commercial finite element method (FEM) software (ANSYS HFSS, R17.0, Ansys, Inc., Canonsburg, PA, USA). The thickness of the glass substrate of the SIW is decided to be 350 μm here, considering ease of wafer handling during the fabrication process. As a glass substrate, BOROFLOAT^®^ 33 glass wafer (SCHOTT AG, Mainz, Germany) whose dielectric constant and loss tangent are 4.6 and 0.0037, respectively, is used. A boron-doped, *p*-type low-resistive silicon wafer with a resistivity of 0.01–0.02 Ω cm is used as a substrate for the via core material. A 50-Ω microstrip line is used for feeding lines of the SIW because the electric field orientation and profile of the microstrip line are almost the same as those of the waveguide. Tapered line transformers are connected between the microstrip feedline and waveguide for smooth field matching from the quasi-TEM mode to TE_10_ mode as well as broadband characteristics. Detailed dimensions of the designed SIW are summarized in Table 1.

**Figure 2:**
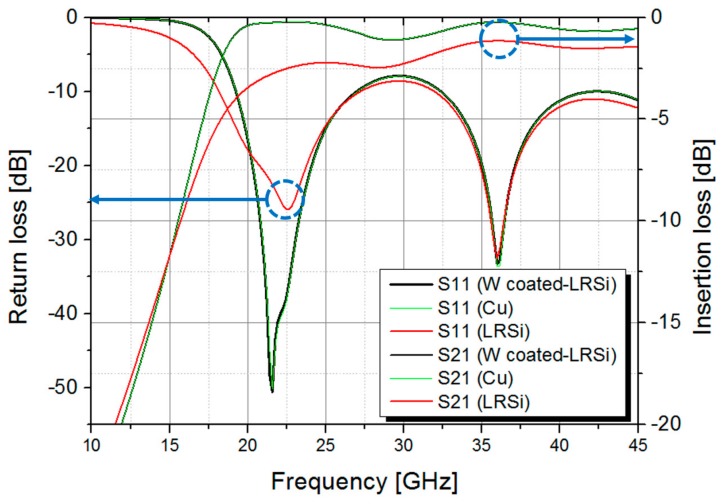
Simulated *S*-parameters of the SIWs with three different via structures: tungsten-coated low-resistive silicon, copper, and pure low-resistive silicon.

Potential of the tungsten-coated silicon via as an alternative for the metal via has been verified by simulating RF performances of the SIWs having the same physical dimensions with three different via materials; tungsten-coated low-resistive silicon, copper and pure low-resistive silicon. Conductivity of the low-resistive silicon is taken to be the inverse of the resistivity of the silicon wafer, and those of the copper and tungsten are set to be 5.813 × 10^7^ S/m and 1.825 × 10^7^ S/m, respectively [22]. As shown in Figure 2, SIWs with tungsten-coated silicon vias and copper vias exhibit almost identical *S*-parameters and the average insertion losses from 20 GHz to 45 GHz, including transitions and feeding lines, are estimated to be 0.54 ± 0.26 dB. The average insertion loss of the SIW with pure low-resistive silicon vias, however, increases significantly compared to both cases, which is 1.88 ± 0.57 dB throughout the same frequency range. It is confirmed from this result that the tungsten-coated silicon vias can effectively replace the metallic copper vias without any degradation in the insertion losses, although the conductivity of tungsten is about three times lower than that of copper.

## 3. Fabrication Process

**Figure 3:**
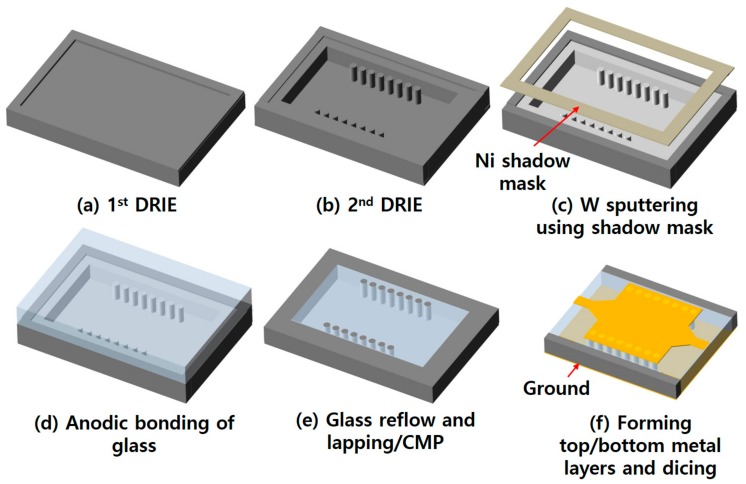
Fabrication process of the SIW with TGSVs.

The overall fabrication process of the SIW is illustrated in Figure 3. The process starts with the first short DRIE of a 4-inch, 525-μm-thick low-resistive silicon wafer using an aluminum mask layer to define a shallow 3-μm-deep rectangular cavity region for the glass reflow. Then the second DRIE is performed inside this cavity to define a 370-μm-deep trench with silicon vias of the same height inside the trench. The size of this trench is designed to be a little bit smaller than that of the first cavity. A 0.6-μm-thick tungsten thin film layer is then selectively coated onto the silicon vias as well as the sidewalls and bottom of the trench by sputtering using a nickel shadow mask. The size of the shadow mask is designed to be smaller than the area of the shallow cavity, so that the top silicon surface required for subsequent bonding of the glass wafer can be protected from metallization by this sputtering process. The top surface of the tungsten-coated silicon vias does not touch the glass substrate during the subsequent anodic bonding process because the height of the silicon vias is slightly lower than the top surface of the silicon wafer because of the first cavity etching.

**Figure 4:**
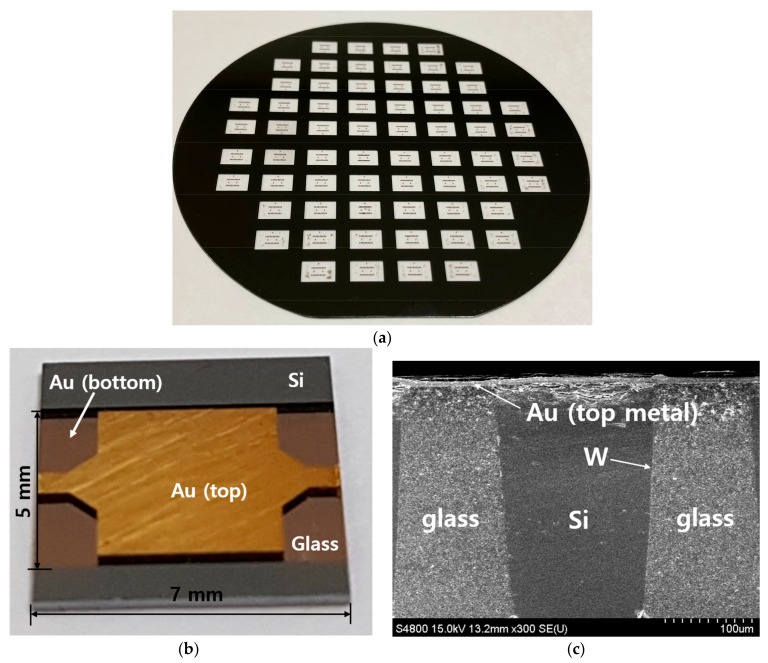
(**a**) Photograph of the wafer after glass reflow and chemical mechanical polishing (CMP) processes; (**b**) Photograph of the fabricated SIW; (**c**) Magnified scanning electron microscope (SEM) image of the silicon via embedded in the glass substrate.

A 4-inch borosilicate glass wafer is then anodically bonded onto this silicon wafer under a vacuum environment. The bonded wafer stack is put into a furnace and heated up to 800 °C with a ramp-up rate of 2 °C/min, kept at that temperature for 8 h, and then cooled down to the room temperature with the same ramp-down rate. During this process, the melted glass is reflowed and filled into the trench due to the pressure difference between the inside of the trench and atmosphere. The overfilled glass on the top surface and part of the silicon are mechanically lapped down and carefully polished precisely using a chemical mechanical polishing (CMP) process until the top surface of the silicon vias is exposed. Remaining silicon and part of the glass at the backside of the wafer are also mechanically lapped and polished down to planarize and set the final thickness of the wafer to be 350 μm. At this stage, both top and bottom surfaces of the silicon vias are exposed. The photograph of the wafer after glass reflow and CMP processes is shown in Figure 4a.

In the next step, both sides of the wafer are completely metallized by sputtering a chrome/gold (25 nm/100 nm) layer. The backside gold layer works as a ground plane of the microstrip feed lines as well as a bottom metal part of the SIW. The top gold layer serves as a seed layer for gold electroplating. On the top side, a 3-μm-thick gold layer is electroplated using a photoresist mold to form the top metal part of the SIW, microstrip feed lines and tapered line transformers. After wet etching of the remaining seed layer, the SIW is finally diced out by cutting out the silicon parts surrounding the glass dielectric substrate. 

The photograph of the fabricated SIW is shown in Figure 4b. The total device size after dicing is 5 mm × 7 mm except for the remaining silicon carrier part in the figure, which can be further cut out without affecting the performances of the device. The magnified cross-sectional scanning electron microscope (SEM) image of the tungsten-coated silicon via embedded in the reflowed glass is shown in Figure 4b. Since the non-ideal property of the DRIE machine we used, silicon via becomes gradually narrower at the bottom of the substrate. Maximum tapered angle of the via about 10° is considered again in the simulation, but it does not affect the performances of the SIW significantly.

## 4. Experimental Results and Discussion

**Figure 5:**
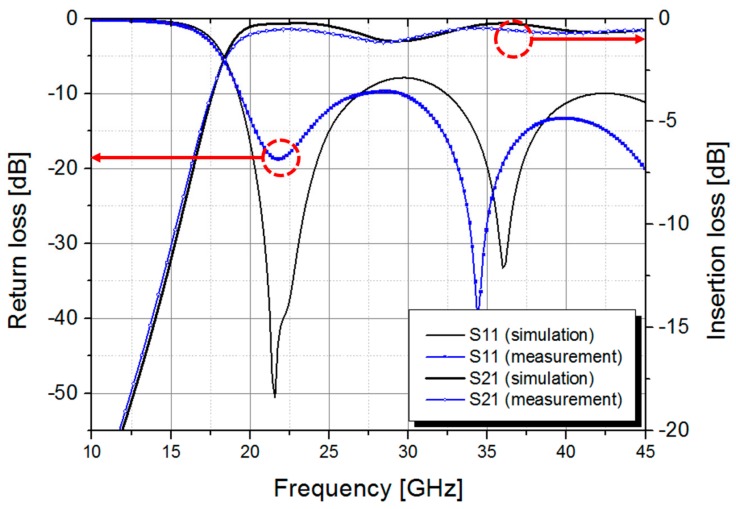
Simulated and measured *S*-parameters of the fabricated SIW.

RF characteristics of the fabricated SIW have been measured by using a commercially available universal text fixture (3680V, Anritsu Corp., Atsugi, Japan) and a HP 8510C vector network analyzer (Keysight Technologies, Santa Rosa, CA, USA) [21]. A standard SOLT (Short-Open-Load-Thru) calibration process is performed with a commercial calibration kit (36804B-10M, Anritsu Corp.) for calibration. The measured *S*-parameters of the fabricated SIW are compared with the simulation results as shown in Figure 5. The 3-dB cut-off frequency of the fabricated SIW is measured to be 17.7 GHz, which is close to the analytical design value of 18.6 GHz. The measured average insertion loss of the fabricated SIW in the range from 20 GHz to 45 GHz is 0.69 ± 0.18 dB with a maximum loss of 1.15 dB occurring around 28 GHz, which is closely matched with the simulation result. The measured return loss is maintained better than 10 dB throughout the same frequency range.

The performances of the fabricated SIW with the proposed tungsten-coated TGSVs have been compared to a couple of reported millimeter-wave SIW results, including our previous micromachined versions, as presented in Table 2. The total length of each SIW is noted for comparison of the insertion losses. The result of this work is showing comparable performances in terms of an insertion loss compared to the SIW with gold-coated silicon vias and a very low loss BCB polymer (with a loss tangent of 0.0008 @ 10 GHz) dielectrics in [20], and other glass-based SIWs with electroplated copper vias in [21,24].

## 5. Conclusions

In summary, a millimeter-wave SIW operating at Ka-band has been demonstrated using tungsten-coated TGSV structures embedded in the reflowed glass substrate. The proposed process allows us to easily fabricate glass substrates with via structures behaving like metallic vias, without individual drilling of micro holes in a glass and time-consuming metal filling processes. The fabricated SIW shows an average insertion loss of 0.69 ± 0.18 dB from 20 GHz to 45 GHz, which is comparable to other millimeter-wave SIWs based on the micromachining-based fabrication technology. The measured return loss is maintained better than 10 dB throughout the same frequency range, which needs to be improved further for integration. The developed glass interposer substrate can be applied not only to the SIW, but also other various millimeter-wave applications such as inductors, filters or antennas where a glass substrate with conductive via structures are required. The operating frequency of the devices using this technology is expected to be elevated to higher bands since the pattern size and substrate thickness can be further reduced thanks to the precision of the micromaching process.

## Figures and Tables

**Table 1:** Detailed geometric parameters of the design.

Parameter	Dimensions [mm]	Description
*d*	0.3	Diameter of the vias
*p*	0.4	(Center-to-center) pitch between the vias
*w*	4	Width of the SIW
*w_1_*	1.5	Larger width of the tapered transformer
*w_2_*	0.5	Width of the microstrip line
*L*	4.4	Lenth of the SIW
*L*_1_	0.8	Length of the tapered transformer
*L*_2_	0.5	Length of the microstrip line
*h*	0.35	Thickness of the substrate

**Table 2:** Performances of the proposed substrate integrated waveguide (SIW) compared with other millimeter-wave SIWs.

Reference	Dielectric Material (Thickness [μm])	Via Material	Frequency [GHz]	Device Length [mm]	Insertion Loss [dB]
[20]	BCB/400	Au-Coated Si	25–40	12.6 ^1^	<1.4
[21]	Borosilicate Glass/350	Electroplated Cu	20–45	10.0 ^1^	<0.95
[24] ^2^	Boroaluminosilicate Glass/130	Electroplated Cu ^3^	20	N/A	0.67/cm
This work	Borosilicate Glass/350	W-coated Si	20–45	7.0 ^1^	<1.15 (Avg. 0.69 ± 0.18)

^1^ Device length includes all the transitions and feeding lines. ^2^ Only simulation results are shown. ^3^ Cu is deposited on the sidewall of the empty via hole in the glass substrate.
